# Azygos ACA fusiform dilatation: embryological insights from co-existing midline lipoma and callosal malformation

**DOI:** 10.1093/bjrcr/uaag008

**Published:** 2026-03-24

**Authors:** Shigeta Miyake, Yu Iida, Neil V Patel, Timo Krings

**Affiliations:** Department of Neurosurgery, Yokohama City University Graduate School of Medicine, Yokohama, Kanagawa, Japan; Department of Neurosurgery, Yokohama City University Graduate School of Medicine, Yokohama, Kanagawa, Japan; Division of Neurointerventional Radiology, UMass Chan School of Medicine, Lahey Hospital, Beth Israel Lahey Health, Boston, MA, United States; Division of Neurointerventional Radiology, UMass Chan School of Medicine, Lahey Hospital, Beth Israel Lahey Health, Boston, MA, United States

**Keywords:** corpus callosum lipoma, azygos anterior cerebral artery, fusiform dilatation, embryology

## Abstract

We present a case of a tubulonodular-type lipoma of the corpus callosum (LCC) with hypogenesis of the corpus callosum (CC) accompanied by an azygos anterior cerebral artery (ACA) with fusiform dilatation adjacent to the lipoma in an 85-year-old female that was incidentally found during a transient ischaemic attack **(**TIA) workup. The patient had no symptoms of LCC or fusiform ACA dilatation. This case highlights the characteristic imaging findings and morphological changes in the adjacent arteries, particularly those associated with LCC. A literature review of 32 reported cases of LCC confirmed that fusiform dilatation of the adjacent vessels is commonly associated with this abnormality. This study suggests that these vascular changes may arise from the abnormal differentiation of the meninx primitiva, affecting both brain structures and blood vessels. Conservative management of fusiform dilatation is recommended as the prognosis appears favourable, with no reported cases of bleeding from such lesions. These findings underscore the importance of understanding the embryological origins of LCC and associated vascular abnormalities.

## Introduction

Intracranial lipoma (IL) is a rare condition accounting for <1% of all primary intracranial space-occupying lesions.^1^^,^^2^ It has been reported in various intracranial cisterns, although it is mostly found along the midline structures. IL is thought to result from abnormal differentiation of the meninx primitiva during the embryonic period and is often associated with brain malformations of varying severity.^3^ These malformations in adjacent tissues may include brain agenesis-hypoplasia, cortical dysplasia, encephalocele with dysplastic meningeal tissue, and intracranial arterial anomalies.^2^^,^^4^^,^^5^ Since IL develops during the embryonic stage, it can sometimes be diagnosed prenatally via ultrasound and is recognized as a feature of various syndromes, including Goldenhar syndrome, PAI syndrome, Haberland syndrome, encephalocraniocutaneous lipomatosis, and frontonasal dysgenesis.^6^^,^^7^ Imaging studies revealed characteristic findings. On computed tomography (CT), the IL appeared as a low-density area (−40 to −85 Hounsfield units), often with calcification and no contrast enhancement. Magnetic resonance imaging (MRI) findings included high signal intensity on T1-weighted images, low signal intensity with fat suppression, high signal intensity on T2-weighted images, and low signal intensity on T2* images.^3^ While ILs are generally stable in size, they strongly adhere to surrounding tissues and blood vessels, making surgical treatment inadvisable unless there is a significant mass effect.^2^

Lipomas of the corpus callosum (LCC) are the most common form of IL.^8^^,^^9^ Although often asymptomatic, they cause headaches and may be associated with psychomotor retardation or epilepsy. LCCs are morphologically classified into tubulonodular and curvilinear types. The tubulonodular type is located anteriorly, measures >2 cm in thickness, and is often associated with calcifications, corpus callosum (CC) dysplasia, and frontal lobe abnormalities. Conversely, the curvilinear type is located posteriorly, less than 1 cm thick, and extends along the CC, occasionally reaching the splenium, but with minimal brain malformations.^8^ The tubulonodular type of LCC can be associated with an azygos anterior cerebral artery (ACA) as well as CC agenesis or hypogenesis.^3^^,^^10^ Additionally, aneurysm formation and fusiform dilatation of azygos ACA have been reported in a previous case series, although the mechanisms underlying these vascular changes remain poorly understood.^11^

Here, we present the case of tubulonodular-type LCC with hypogenesis of the CC, accompanied by an azygos ACA with fusiform focal dilatation associated with the LCC. This study aimed to characterize the vascular changes associated with LCC based on this case and a review of the literature, as well as to discuss the morphological alterations of the surrounding structures associated with LCC from an embryological perspective. Written informed consent was obtained from the patient for publication of this case review, including accompanying images.

## Case presentation

**Figure 1:**
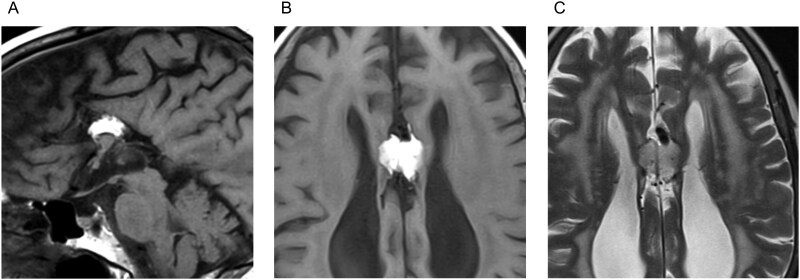
The MRI features of an 85-year-old female with congenital corpus callosum hypogenesis. The medial sagittal plane of the FLAIR image (A) shows hypogenesis of the corpus callosum with lipoma of the corpus callosum. T1-weighted axial images (B) and T2-weighted axial images (C) show colpocephaly and a tubulonodular-type lipoma of the corpus callosum.

**Figure 2:**
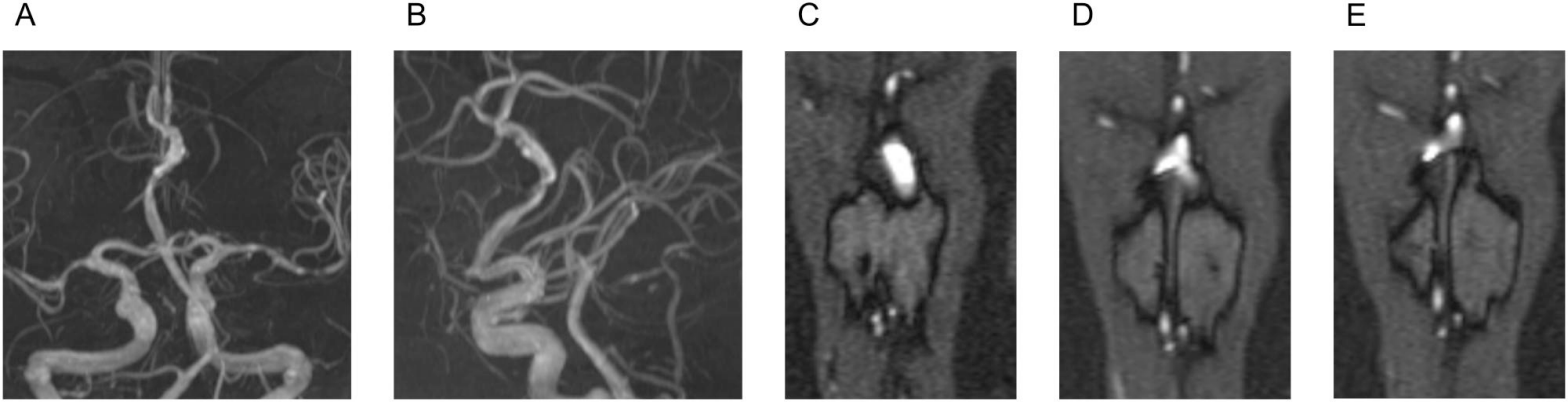
MRA revealed atherosclerotic stenosis in the middle cerebral artery regions and evidence of an azygos anterior cerebral artery with fusiform dilatation (A and B). Time-of-flight imaging showed no evidence of dissection in the fusiform dilatation. Instead, fusiform dilatation was observed exclusively in the region adjacent to the lipoma (C-E).

An 85-year-old high-functioning female with a medical history of hypertension, hyperlipidemia, right cranial nerve IV palsy, and a transient ischaemic attack (TIA) was referred to our vascular clinic for the evaluation of an arterial anomaly that was incidentally found during the TIA workup. CT and MRI revealed hypogenesis of the CC with associated colpocephaly and a tubulonodular-type anterior midline LCC (Figure 1). No prior cranial imaging was available, and LCC had not been previously identified. Magnetic resonance angiography (MRA) revealed mild intracranial atherosclerosis and an azygos ACA with fusiform dilatation adjacent to the LCC (Figure 2A and B). Time-of-flight imaging revealed no imaging findings suggestive of dissection, such as an intimal flap, or a saccular aneurysm associated with the fusiform dilatation (Figure 2C-E).

As the patient had no symptoms related to LCC or ACA fusiform dilatation, management focused on controlling vascular risk factors and conservative management of the ACA fusiform dilatation. She has remained asymptomatic for the past 4 months.

## Literature review

A narrative literature review was conducted in January 2025 using the PubMed database, employing combinations of the English-language keywords “intracranial lipoma,” “arterial anomaly,” “aneurysm,” “fusiform dilatation,” “fusiform ectasia,” and “artery.” After excluding duplicate reports, case reports and review articles describing arterial anomalies adjacent to the IL were analysed. A total of 32 cases of IL with aneurysmal arterial change^4^^,^^5^^,^^12^^,^^13^ and 6 cases of LCC with azygos ACA,^10–12^^,^^14^^,^^15^ including the present case, were reviewed (Tables 1 and 2).

**Table 1:** Literature review of aneurysmal change adjacent with intracranial lipoma.^4^^,^^5^^,^^12^^,^^13^

Publication		Patients		Location of lipoma	Aneurysmal change		
Publish year	Case number	Age	Sex		Affected vessel	Saccular	Fusiform dilatation
1975	21	–	–	Corpus callosum	ACA	2	19
1986	1	53	Female	Corpus callosum	ACA	1	
1989	1	52	Female	Corpus callosum	ACA	1	
1992	1	26	Female	Sylvian	MCA	1	
2005	1	27	Male	Sylvian	MCA	1	
2005	1	50	Female	Sylvian	MCA	1	
2006	1	55	Female	Sylvian	MCA	1	
2013	1	60	Female	Sylvian	MCA	1	
2018	1	11	Male	Sylvian	MCA		1
2023	1	42	Female	Sylvian	MCA		1
2023	1	80s	Male	Interpeduncular fossa	PCA	1	
Current	1	85	Female	Corpus callosum	ACA		1

Abbreviations: ACA = anterior cerebral artery; MCA = middle cerebral artery; PCA = posterior cerebral artery.

**Table 2:** Literature review of the cases with azygos ACA with LCC.

Publish year	Age	Sex	Type of LCC	Hypogenesis of the CC	Aneurysmal change	Haemorrhage
1972^11^	21	Male	Tubulonodular	Yes	Fusiform dilatation	None
1972^14^	15	Male	Tubulonodular	Yes	None	None
1975^12^	51	Male	–	–	Fusiform dilatation	None
1986^15^	53	Female	–	Yes	Saccular	Yes
2021^10^	57	Female	Tubulonodular	Yes	None	None
Current	85	Female	Tubulonodular	Yes	Fusiform dilatation	None

Abbreviations: ACA = anterior cerebral artery; CC = corpus callosum; LCC = lipoma of the corpus callosum.

Among the reviewed cases, 24 reported that arterial changes in the ACA were associated with LCC. The age of the patients varied (mean: 49.2, range: 11-85 years), and the cases demonstrated a female predominance (72.7%). Among aneurysmal changes, saccular aneurysms were more frequently observed in non-ACA regions, reported in 6 of 8 cases (75.0%), while fusiform dilatation was the most common vascular change in the ACA, reported in 20 of 24 cases (83.3%, *P* < .01, chi-square test). Seven cases described aneurysmal changes in the middle cerebral artery (MCA) (5 cases of the saccular type and 2 cases of the fusiform type) associated with lipomas of the Sylvian fissure, and 1 case involved saccular aneurysmal changes in the posterior cerebral artery associated with a lipoma of the interpeduncular cistern (Table 1).

No trends were observed in age or sex in the cases of LCC with azygos ACA. All patients showed hypogenesis of CC and tubulonodular-type LCC. Furthermore, aneurysmal arterial changes were associated with LCC and azygos ACA in 4 of 6 patients (66.7%). Only one case of saccular-type aneurysm was reported to develop bleeding; however, no bleeding was reported in the case of fusiform dilatation (Table 2).

## Discussions

In this case report and review, we describe an exceedingly rare association between hypogenesis of the CC and LCC, azygos ACA, and fusiform dilatation of the artery adjacent to the LCC. While LCC is associated with hypogenesis of the CC in 68.8% of patients with LCC^3^ and 2.1% of patients with a− or hypogenesis of the CC,^16^ only 5 cases of LCC with azygos ACA have been reported. A− or hypogenesis of the CC is associated with 5.3% of patients with azygos ACA,^17^ and arterial anomalies are associated with 66.7% of LCC patients with an azygos ACA (Table 2). A major limitation of this review is the potential for selection bias, as the extreme rarity of arterial anomalies associated with LCC precludes comprehensive population-based analysis. However, an association among all 3 findings has only been reported in 1 patient. We believe that, *albeit* rarely, this association may shed light on a common embryological cause and, in a broader sense, on the association between brain and vascular development, which should be regarded as a parallel and interconnected process rather than 2 separate developments.

### Embryological hypothesis

Meninx primitiva, the precursor tissue of IL,^3^ is composed of mesodermal and neural crest cells. Meninx primitiva is initially responsible for the extrinsic diffusion of nutrients in neural tissue, but as the neural tissue develops, it mediates the growth of the brain and surrounding tissues and eventually becomes supportive tissue.^18^^,^^19^ Specifically, it appears between the 5th and 7th weeks of gestation as a membrane surrounding neural tissue, which later contributes to the skull, meninges, brain surface, as well as to the intracranial vessel wall.^19^ This developmental origin explains the extension of LCC into the skull and subcutaneous areas,^9^ as well as the possibility of concomitant malformations of the adjacent cerebral cortex and blood vessels.^2^^,^^4^^,^^5^ Pathological studies of the cerebral cortex adjacent to ILs have revealed mixed findings of mesenchymal and neural tissue,^20^ suggesting that the meninx primitiva may play a role in the orderly development of astrocytes, neurons, and synapses through its interaction with neural tissue during the embryological period.

The embryological roles of meninx primitiva in brain development have been described through both direct and indirect mechanisms.^19^ It directly influences brain tissue development by contributing to the formation of pial basement membranes, neuronal migration and differentiation, and the maintenance of neural stem cells. Specifically, in the development of CC, meninx primitiva has been reported to inhibit axon outgrowth via the secretion of BMP7, which is critical for the formation of normal axonal bundles. Indirectly, the meninx primitiva plays a vital role in cerebral vascularization by forming an outer vascular layer and constructing perforating vessels. Cerebral hypoxic signalling is essential for the development of vascular structures during early brain development, particularly between the 5th and 7th weeks of gestation.^18^ Pathologically, the abnormal activation of hypoxic signalling has been associated with corticoarterial malformations.^21^^,^^22^ Dysfunction of the meninx primitiva can also result in hypo- or agenesis of the neural tissue due to insufficient vascularization, which fails to adequately support the hypoxic signalling requirements of the brain.

The CC is a neural structure that connects the cerebral hemispheres bilaterally. Its development begins around the 11th to 12th weeks of gestation, with its full length formed by 20 weeks, and further maturation continues until 30 weeks of gestation.^23^ CC formation progresses in an anterior-to-posterior direction, and the characteristic anterior protrusion of the genus is typically not visible before 14 weeks of foetal development.^24^ Agenesis of the CC can result from a variety of factors, including metabolic (eg, alcohol exposure) and genetic causes.^24^ In the context of LCC, it is thought to result from a lack of meningeal scaffolding that supports axonal elongation and the orderly development of neuronal structures.

In tubulonodular-type LCC,^8^ the concomitant a− or hypogenesis of the CC may depend on whether the meninx primitiva differentiation abnormality occurs before or after the 11th or 12th weeks of gestation, when CC formation begins.^23^ Conversely, in curvilinear-type LCC,^8^ CC formation is typically preserved, and the lipoma elongates along the CC. This suggests that abnormalities in meninx primitiva differentiation in the curvilinear LCC occur later in the embryonic period (12-20 weeks), when the CC elongates posteriorly.^24^

CC agenesis is associated with azygos ACA formation.^17^ Azygos ACA is a rare variant, occurring in approximately 1% of cases,^25^ and is thought to arise from the abnormal fusion of the median branch of the primitive olfactory artery or persistence of a median artery in the CC.^26^ The median artery typically forms around 9 weeks of gestation and regresses with the development of the CC. In cases of CC agenesis, the median artery fails to regress, leading to the formation of azygos ACA. Comparative anatomy supports this relationship. In species with smaller CC volume such as monkeys^27^ and dogs,^28^ azygos ACAs are more commonly observed.^29^ This correlation underscores the role of CC development in the formation of the normal ACA anatomy; conversely malformations of the CC will lead to a “dysmaturation” of the ACA which thus remains in a more primitive or earlier embryological state. The percentage of azygos ACA accompanying LCC is unknown because few reports have focused on the relationship between LCC and ACA morphology (Table 2). However, considering this embryological background, the reports to date may have underestimated the true incidence. Considering the timing of meninx primitiva differentiation abnormalities and the morphology of LCC, it appears that CC agenesis facilitates the phenotypic expression of the azygos ACA.

### Morphological change of ACA

In the present study, fusiform dilatation was identified as the predominant ACA change associated with LCC. Fusiform aneurysms are generally rare in the distal ACA, with an incidence of only 1 in 108 cases of ACA aneurysms.^30^ Pathologically, non-traumatic fusiform aneurysms of the ACA are increasingly recognized not as isolated spontaneous malformations, but as the sequelae of subclinical arterial dissections undergoing maladaptive vascular remodelling.^31^ However, in the present case, there was no evidence of dissection, suggesting an alternative aetiology. While aneurysm formation is frequently reported in azygos ACAs, these are predominantly saccular aneurysms that arise at the branching point of the azygos ACA.^25^^,^^26^ As pointed out earlier, morphological changes in arteries adjacent to ILs have been reported,^4^^,^^5^ with saccular aneurysms being more common except in the ACA. These observations suggest that the characteristic fusiform dilatation of the ACA associated with LCC results from a combination of factors, including vascular structure, hemodynamics, and lesion length.

From a structural perspective, the meninx primitiva forms the arachnoid and the outer layer of the cerebral arteries,^19^^,^^32^ and its loss may lead to structural fragility. This fragility may explain the increased aneurysm formation in the arteries adjacent to the ILs. Hemodynamic stress as well as the inflammatory mechanisms underlying aneurysm formation,^25^^,^^26^ could further contribute to morphological changes in the vessels. Additionally, tubulonodular-type LCCs are thought to develop early during embryogenesis, during which the ACA is elongated.^23^^,^^24^ The presence of the lipoma during this period may have led to an increase in its size along with the ACA and an extended contact area with the ACA. In our case, the fusiform dilatation of the ACA corresponded to the region bordering the lipoma.

Thus, LCC may influence the development of both the CC and ACA, leading to a− or hypogenesis of the CC, and secondarily to an azygos ACA. Whether the additionally observed fusiform dilatation is related to (1) hemodynamic issues related to the altered flow pattern in an azygos ACA, (2) mechanical/structural alterations due to the LCC adhering to the vessel wall, or (3) an underlying altered common pathway of brain and vessel development, as hypothesized in the formation of cortical dysplasia associated with DVAs, remains unknown.

Irrespective of the underlying mechanism of fusiform dilatation, the natural history of these arterial anomalies is likely to be benign. A literature review did not report bleeding from fusiform dilatation adjacent to the ILs.^4^^,^^5^^,^^12^^,^^13^ Only cases involving ruptured MCA saccular aneurysms not in contact with the LCC,^4^ or clipping of ruptured saccular type aneurysms bordering ILs,^13^ have been described. These findings suggest that the prognosis of fusiform dilatation in ACA associated with LCC is favourable. Furthermore, fusiform dilatation of the carotid artery following surgery for paediatric suprasellar tumours served as a useful comparison.^33^^,^^34^ This rare condition, which occurs in approximately 10% of cases after tumour resection, is attributed to mechanical damage to the internal carotid artery during tumour removal. The similarity between fusiform dilatation caused by outer membrane damage during the cerebrovascular development period^34^ and ACA morphological changes in LCC highlights a possible shared mechanism. Given the absence of reported cases of bleeding from fusiform dilatation adjacent to the ILs, and the uneventful clinical course of fusiform dilatation of the carotid artery,^34^ conservative management of ACA fusiform dilatation associated with LCC is advisable. Imaging follow-up is recommended in accordance with standard practices for unruptured intracranial aneurysms, and prompt imaging evaluation may be recommended if any neurological changes occur.

## Conclusions

In this study, we described the characteristic clinical presentation of CC hypogenesis and azygos ACA with fusiform dilatation in patients with LCC. The dysplasia of adjacent tissues complicating ILs resulting from abnormalities in meninx primitive differentiation is characterized by both location and timing and can be explained from an embryological perspective. Based on this embryological understanding, we postulated that the failure of differentiation of the meninx primitive around the 10th week of gestation leads to the formation of an LCC that inhibits normal CC development, thus leading to the failure of normal ACA development. The fusiform dilatation observed in association with LCC may be related to hemodynamic, structural, or developmental causes and is likely to have a benign natural history.

## Learning points

- Lipomas of the corpus callosum (LCC) often coexist with developmental anomalies such as hypogenesis of the corpus callosum and arterial variants like azygos ACA.
- Fusiform dilatation of the ACA adjacent to LCC represents a benign, non-aneurysmal vascular change likely due to abnormal meninx primitiva differentiation rather than dissection.
- Understanding the embryological link between neural and vascular structures helps explain concurrent malformations in brain parenchyma and arteries.
- Conservative management is generally appropriate, as no haemorrhagic cases of fusiform dilatation associated with LCC have been reported.
